# Growth factors regulate proteinase activated receptor – 2 (PAR-2) on airway epithelium

**DOI:** 10.1186/1710-1492-10-S2-A11

**Published:** 2014-12-18

**Authors:** Vivek Gandhi, Drew Nahirney, Harissios Vliagoftis

**Affiliations:** 1 Pulmonary Research Group, Department of Medicine, University of Alberta, Edmonton, Alberta, T6G 2S2, Canada

## Background

Many aeroallergens activate PAR-2 receptors on the airway epithelium. We have shown that PAR-2 activation participates in allergic sensitization and allergic airway inflammation in animal models of asthma. Moreover, PAR-2 is upregulated on the airway epithelium of asthmatics, but the mechanisms and factors responsible are unknown. As asthmatic airways are under various types of physiological stress, we hypothesized that cellular stress upregulates PAR-2 on airway epithelium and this upregulation is functional.

## Methods

Human bronchial epithelial cells were cultured with or without growth factors for 24hrs/48hrs and PAR-2 mRNA levels were studied by qRT-PCR. PAR-2 functions were assessed by measuring PAR-2-mediated calcium release from intracellular stores into the cytoplasm and IL-8 release in supernatants.

## Results

We have previously shown that growth factor deprivation, but not oxidative stress or hypoxia, significantly upregulates PAR-2 mRNA in normal bronchial epithelial cells. We now show that growth factor deprivation also induces PAR-2 upregulation in airway epithelial cells from asthmatic individuals (2.1 +/- 0.1 fold, n=2); this upregulation was reversible upon growth factor addition. We omitted individual growth factor from the culture media. Omission of only insulin caused significant PAR-2 mRNA upregulation (1.6 +/- 0.1 fold, n=4). In addition, supplementation of stressed cells with insulin reversed the growth factor deprivation-induced PAR-2 upregulation (n=3). PAR-2-mediated activation of stressed cells induced more calcium release from internal stores and higher release of the inflammatory mediator IL-8 (2.1 +/- 0.2 fold, n=6) compared to non-stressed cells.

## Conclusions

Growth factor deficiency could be the driving force for PAR-2 upregulation in asthmatic airways. Insulin may be one of the growth factors that regulate PAR-2 transcription. In conditions of growth factor deprivation, PAR-2 upregulation may lead to increased activation of airway epithelial cells resulting in the higher release of pro-inflammatory mediators. Therefore, understanding PAR-2 regulation may allow the development of new anti-inflammatory approaches for airways diseases.

